# Loneliness, cerebrovascular and Alzheimer's disease pathology, and cognition

**DOI:** 10.1002/alz.14196

**Published:** 2024-09-05

**Authors:** Patrick Lao, Christina B. Young, Chima Ezeh, Bayardo Lacayo, Dominika Seblova, Ryan M. Andrews, Laura Gibbons, A. Zarina Kraal, Indira Turney, Kacie D. Deters, Vonetta Dotson, Jennifer J. Manly, Lisa L. Barnes, Laura B. Zahodne

**Affiliations:** ^1^ Department of Neurology Columbia University New York New York USA; ^2^ Department of Neurology and Neurological Sciences Stanford University School of Medicine Stanford California USA; ^3^ Second Faculty of Medicine Charles University Prague Prague Czech; ^4^ Department of Epidemiology Boston University Boston Massachusetts USA; ^5^ Department of Biometry and Data Management Leibniz Institute for Prevention Research and Epidemiology—BIPS Bremen Germany; ^6^ General Internal Medicine School of Medicine University of Washington Seattle Washington USA; ^7^ Department of Integrative Biology and Physiology College of Life Sciences University of California Los Angeles Los Angeles California USA; ^8^ Department of Psychology and Gerontology Institute Georgia State University Atlanta Georgia USA; ^9^ Rush Alzheimer's Disease Center Rush University Medical Center Chicago Illinois USA; ^10^ Department of Psychology University of Michigan Ann Arbor Michigan USA

**Keywords:** Alzheimer's disease pathology, autopsy, cognition, cognitive reserve, infarcts, inverse odds of selection weights, loneliness, microinfarcts, transportability

## Abstract

**INTRODUCTION:**

Loneliness has a rising public health impact, but research involving neuropathology and representative cohorts has been limited.

**METHODS:**

Inverse odds of selection weights were generalized from the autopsy sample of Rush Alzheimer's Disease Center cohorts (*N* = 680; 89 ± 9 years old; 25% dementia) to the US‐representative Health and Retirement Study (*N* = 8469; 76 ± 7 years old; 5% dementia) to extend external validity. Regressions tested cross‐sectional associations between loneliness and (1) Alzheimer's disease (AD) and cerebrovascular pathology; (2) five cognitive domains; and (3) relationships between pathology and cognition, adjusting for depression.

**RESULTS:**

In weighted models, greater loneliness was associated with microinfarcts, lower episodic and working memory in the absence of AD pathology, lower working memory in the absence of infarcts, a stronger association of infarcts with lower episodic memory, and a stronger association of microinfarcts with lower working and semantic memory.

**DISCUSSION:**

Loneliness may relate to AD through multiple pathways involving cerebrovascular pathology and cognitive reserve.

**Highlights:**

- Loneliness was associated with worse cognition in five domains.
- Loneliness was associated with the presence of microinfarcts.
- Loneliness moderated cognition–neuropathology associations.
- Transportability methods can provide insight into selection bias.

## 1 BACKGROUND

Alzheimer's disease (AD) pathology, the most common cause of dementia (60%–80%^1^, ^2^), is defined pathologically by sufficient amyloid beta (Aβ) plaque^3^ and neurofibrillary tau tangle burden^4^ in specific brain regions that precede neurodegeneration and cognitive impairment,^5^, ^6^, ^7^ particularly in episodic memory. However, ≈30% of individuals with sufficient amyloid and tau pathology do not develop AD dementia^8^ and ≈40% of dementia cases could be prevented by intervention on modifiable risk factors (i.e., education, hypertension, hearing impairment, smoking, obesity, depression, physical inactivity, diabetes, and low social contact).^9^ Other disease biomarkers (e.g., cerebrovascular disease and neuroinflammation) should be considered in relation to these modifiable risk factors (e.g. low social contact), elevated amyloid and tau pathology, cognitive impairment, and/or the relationship between pathology and cognitive impairment (i.e., cognitive reserve).^10^, ^11^, ^12^

Loneliness,^13^ social engagement,^14^, ^15^ and other related constructs^16^, ^17^, ^18^ have been implicated consistently in cognitive decline and incident AD dementia.^9^, ^19^, ^20^, ^21^ Emotional loneliness is a distressing feeling from the lack of satisfaction with social connections and can occur in the context of large social networks.^22^ The psychological stress associated with chronic emotional loneliness may operate along different pathways compared to the resources and opportunities provided by social interactions.^16^ Loneliness and related measures worsened from 2003 to 2020,^23^ and were declared an epidemic in the United States in 2023.^16^ The effect of loneliness on all‐cause premature death was approximately equal to that of smoking, obesity, and physical inactivity.^24^ Loneliness had the strongest associations with heart disease (29%) and stroke (32%) risk^25^ and further associations with treatment adherence and hospital readmission.^26^, ^27^, ^28^ Biologically, the associations of loneliness with inflammation^29^ and vascular health^30^, ^31^, ^32^ may represent common pathways for a wide range of disease outcomes. Social relationships influence inflammation in early life to the same extent as physical activity, whereas social isolation increase the risk of hypertension in later life to a greater extent than diabetes.^33^ Social contact may encourage beneficial health behaviors or provide cognitive stimulation, and reduced social contact may account for 4% of preventable dementia cases.^9^ A study from the Rush Alzheimer's Disease Center cohorts with pathological confirmation demonstrated that greater loneliness was not associated with AD or cerebrovascular pathology, but was associated with faster cognitive decline over time and risk for incident AD dementia.^13^ Furthermore, these associations were independent of depression and social isolation.^13^

Thoroughly characterized autopsy samples are well‐suited to address the association of loneliness with pathology with higher sensitivity, specificity, and spatial resolution compared to in vivo methods; however, individuals who agree to brain donation may be a select group,^34^ and individuals who endorse loneliness may be less likely to participate in research. Transportability methods^35^, ^36^ weight a selected sample to approximate an external population‐representative sample to generalize results. Therefore, we sought to improve the external validity of analyses that examined how loneliness, as a psychosocial stressor, is associated with two of the most common pathologies underlying dementia, and how those biological correlates may be associated with the primary outcome for dementia risk—cognitive performance. We hypothesized that greater loneliness would be associated with (1) greater AD and cerebrovascular pathology, (2) greater cognitive impairment across all domains independently of pathology, and (3) a stronger negative association between pathology and cognition, reflecting lower cognitive reserve. Given that loneliness may be more present in the overall U.S. population compared to autopsy samples, we also predicted that these associations would be stronger after transporting models.

## 2 METHODS

### 2.1 Autopsy data

Participants with neuropathological data were drawn from the Rush Alzheimer's Disease Center, which includes the Religious Orders Study, the Rush Memory and Aging Project, and the Minority Aging Research Study (ROS/MAP/MARS^37^, ^38^; *N* = 680; 89 ± 9 years old; 25% with dementia). Full information about the study participants and variables can be found at the Rush Alzheimer's Disease Center Research Resource Sharing Hub (https://www.radc.rush.edu/documentation.htm). All participants provided informed consent and all study procedures were approved by the Rush University Institutional Review Board and performed according to the Declaration of Helsinki.

The National Institute on Aging‐Reagan (NIA‐Regan) rating scale^39^ combines the Consortium to Establish a Registry for Alzheimer's Disease (CERAD) rating of amyloid (modified for no age or clinical diagnosis adjustment) and the Braak staging of tau into a single rating of AD (none: no CERAD, Braak 0; low likelihood: infrequent CERAD/possible AD, Braak I‐II; moderate likelihood: moderate CERAD/probable AD, Braak III‐IV; high likelihood: frequent CERAD/definite AD, Braak V‐VI). For these analyses, NIA‐Reagan ratings were collapsed into none/low likelihood and moderate likelihood/high likelihood of AD due to the distribution in this sample.^40^ The presence of chronic infarcts and the presence of chronic microinfarcts across the whole brain were collapsed into “Not present” or “Present” due to their skewed distributions.^41^, ^42^

### 2.2 Loneliness data

Loneliness was evaluated with five items from a modified version of the De Jong‐Gierveld Loneliness Scale to capture loneliness or the feeling of being disconnected from others.^13^ Items included “I experience a general sense of emptiness”, “I miss having people around”, “I feel like I don't have enough friends”, “I often feel abandoned”, and “I miss having a really close friend”. Each item was rated from 1 to 5 on a Likert scale and the average of the five items was calculated, with higher values representing greater loneliness. A modified, 9‐item version of the Center for Epidemiologic Studies Depression scale (CES‐D) removed the “I feel lonely” item to adjust for unique associations involving depressive symptoms (as a continuous variable) compared to loneliness, with higher values representing greater depressive symptoms. Loneliness and depressive symptoms were collected at each study visit. Given the relative stability of loneliness in the Rush cohort over time^13^ and to align with our goal of assessing associations with pathology, we used loneliness and depressive symptoms at the last visit.

### 2.3 Cognitive data

Cognition was assessed with a comprehensive neuropsychological battery at each visit. We used domain scores at the last visit. Domain scores^43^ were calculated by converting raw scores on each cognitive test to *z*‐scores, using the baseline visit mean and SD for all available participants, and then averaging the *z*‐scores. Higher *z*‐scores correspond to better performance across all domains. The episodic memory composite included Word List learning, Word List recall, Word List recognition, East Boston immediate recall, East Boston delayed recall, Logical Memory immediate recall, and Logical Memory delayed recall. The working memory composite included the Digit Span forward, Digit Span backward, and Digit Span sequencing. The semantic memory composite included the 15‐item Boston Naming Test, Animal and Fruit/Vegetable category fluency, and word reading as assessed by the 10‐item National Adult Reading Test (NART) for ROS and MAP or the 15‐item Wide Range Achievement Test (WRAT) for MARS. The perceptual orientation (i.e., visuospatial ability) composite included the Line Orientation test and the 16‐item Progressive Matrices Test. The perceptual speed composite included the oral Symbol Digits Modality test, Number comparison test, Stroop color naming, and Stroop word reading.

**RESEARCH IN CONTEXT**

1. **Systematic review**: The authors reviewed the literature for Alzheimer's disease (AD), cerebrovascular disease, and loneliness using traditional sources (e.g., PubMed), included the U.S. Surgeon General's Our Epidemic of Loneliness and Isolation.
2. **Interpretation**: The current study demonstrates that loneliness impacts AD through cerebrovascular disease, rather than amyloid and tau pathology, and its associations with cognition using autopsy data. Inverse odds of selection weighting transports findings from self‐selected samples with brain donation to U.S. population representative samples.
3. **Future directions**: Neuroimaging and plasma‐based biomarker studies earlier in the life course can provide additional insight into how loneliness affects the development and progression of AD and related dementias. Inclusion of minoritized groups, which experience higher rates of loneliness, can provide further insight into how these mechanisms operate at different levels of exposure.

### 2.4 Statistical methodology

To understand the distribution of loneliness across demographic characteristics, we assessed general linear models with the following general specifications: Loneliness ∼ Age + Sex + Race + Education + Depressive symptoms. A series of statistical models tested the three main hypotheses (main effect of loneliness on AD and cerebrovascular pathology; main effect of loneliness on cognitive performance; and interaction of loneliness and pathology on cognitive performance). For the first hypothesis, logistic regression models had the following general specifications: Neuropathological Rating ∼ Loneliness + Other Neuropathological Ratings (i.e., those that were not included as the dependent variable) + Age + Sex + Race + Education + Depressive symptoms + Time between the last visit and autopsy. This was done to account for mechanistic links between different types of cerebrovascular disease, amyloid, and tau.^44^ For the second and third hypotheses, general linear models had the following general specifications: Cognitive domain score ∼ Each Neuropathological Rating + Loneliness + Each Neuropathological Rating × Loneliness + Age + Sex + Race + Education + Depressive symptoms + Time between the last visit and autopsy. Cognitive domain scores, loneliness, and time‐varying covariates (i.e., age, depressive symptoms) were taken from the last visit, and the time between the last visit and autopsy was included as an additional covariate. Time‐invariant covariates included sex, race, and years of education. Each cognitive domain score was the outcome in its own model. All neuropathological ratings were additively tested in a single cognitive domain model (5 models); results did not diverge from individual neuropathological rating and cognitive domain scores (3 Neuropathological Ratings × 5 Cognitive Domains = 15 models).

The transportability (i.e., the generalizability of findings from a sample to an external population) of the associations of interest were tested using inverse odds of selection weights (IOSWs) derived from the Health and Retirement Study (HRS). The HRS is a nationally representative longitudinal survey including >37,000 individuals older than age 50 in 23,000 households in the United States.^45^ Variables hypothesized to be related to selection into the sample (i.e., ROS/MAP/MARS) and the external population (i.e., HRS) were harmonized. These variables included demographics, socioeconomic status, measures of general health, and cognition. If harmonized variables were missing, multiple imputation was performed in the sample and external population, separately. Then, IOSWs were calculated such that the sample and external population achieved covariate balance (i.e., within 0.25 SD on all harmonized variables after weighting was considered approximately equal). The simplest model from an iterative process was chosen to derive IOSW weights, which were then stabilized to maintain original analytic sample size and trimmed to remove outliers beyond the 99th percentile. All harmonized variables were checked for balance, regardless of whether they were included in the IOSW model, to ensure that weights were not overfit. Finally, IOSWs were applied to the series of general linear models testing Aims 1–3 such that the final estimates reflect the associations weighted to the U.S. population. For example, ROS/MAP/MARS participants with pathological data were older compared to HRS participants; with weighting, these younger individuals were up‐weighted/over‐represented in the model, emphasizing the strength and direction of the associations of interest at a younger age range compared to an older one. Similarly, with weighting there was a lower proportion of individuals with AD pathology, in line with the younger age of the weighted sample. Therefore, weighted results may extend external validity and capture associations relevant to early‐stage development of pathology rather than late‐stage pathology. It is important to note that disease and cognitive reserve mechanisms may differ based on location along the AD continuum. Details about harmonization, multiple imputation, and the IOSW model can be found in the Supplementary Materials. All statistics were run in R (version 4.0.2).

## 3 RESULTS

Participants with observed autopsy data (*N* = 680) were on average 89 years of age at death, mostly women (68%), mostly White (94%), and had 15 years of education (Table 1). As expected, the IOSW weighted data, which approximates the U.S. population 65 years of age or older, was slightly younger, mostly women, had more Black participants, and had slightly fewer years of education. Loneliness was not different after IOSW weighting, but depressive symptoms increased slightly (Table 1). AD pathology was 56% moderate/high likelihood in the observed sample, but decreased to 15% in the IOSW weighted data (Table 1). Infarcts and microinfarcts were not as different after IOSW weighting, whereas mean cognitive performance across all domains (except for visuospatial ability) increased after IOSW weighting (Table 1).

**TABLE 1:** Demographic, exposure, and outcome variables in the observed data and weighted data using inverse odds of selection weights.

	Observed	Weighted
	*N* = 680	Inverse odds of selection weights (ISOWs) = 1.3 ± 4.4, 0.001 to 38.9
Time between last visit and autopsy	1.0 ± 1.3, 0.01 to 9.3	0.9 ± 1.1, 0.01 to 9.3
Age	88.8 ± 6.5, 63 to 107.9	84.3 ± 8.8, 63 to 107.9
Sex	220 (32%) men, 460 (68%) women	244 (36%) men, 436 (64%) women
Race	637 (94%) White, 43 (6%) Black	578 (85%) White, 102 (15%) Black
Education	14.8 ± 2.9, 5 to 28	13.2 ± 3, 5 to 28
Loneliness	2.4 ± 0.6, 1 to 5	2.4 ± 0.7, 1 to 5
Depressive symptoms	1.0 ± 1.4, 0 to 7	1.5 ± 1.9, 0 to 7
AD pathology	297 (44%) no AD/low likelihood, 383 (56%) intermediate/high likelihood	578 (85%) no AD/low likelihood, 102 (15%) intermediate/high likelihood
Chronic Infarcts	444 (65%) not present, 236 (35%) present	460 (68%) not present, 220 (32%) present
Chronic Microinfarcts	467 (69%) not present, 213 (31%) present	491 (72%) not present, 189 (28%) present
Episodic Memory	−0.5 ± 1.1, −3.6 to 1.5	−0.1 ± 1.2, −3.6 to 1.5
Working Memory	−0.4 ± 0.8, −3.4 to 1.9	−0.2 ± 1.1, −3.4 to 1.9
Semantic Memory	−0.7 ± 1.1, −8 to 2	−0.4 ± 1.4, −8 to 2
Visuospatial ability	−0.2 ± 0.9, −3.2 to 1.7	−0.2 ± 1.2, −3.2 to 1.7
Perceptual Speed	−0.9 ± 0.9, −3.2 to 1.3	−0.8 ± 1.1, −3.2 to 1.3
Dementia	510 (75%) without dementia diagnosis, 170 (25%) with dementia diagnosis	582 (85%) without dementia diagnosis, 98 (15%) with dementia diagnosis

*Note*: Mean and SD as well as the range are provided for continuous variables. The count and percentage are provided for categorical variables. IOSWs below 1 indicate that an individual is down‐weighted in the model and weights above 1 indicate that an individual is up‐weighted in the model.

### 3.1 Demographics, loneliness, and pathology

In the observed data (i.e., the unweighted ROS/MAP/MARS cohort), older age, being a man, lower education, and greater depressive symptoms were associated with greater loneliness. After IOSW weighting, only older age and greater depressive symptoms were associated with greater loneliness (Table 2). Older age was associated with AD pathology and microinfarcts; infarcts were positively associated with microinfarcts; and loneliness was not associated with AD pathology, infarcts, or microinfarcts in the observed data (Table 3). After IOSW weighting, older age, self‐identifying as Black, and greater depressive symptoms were associated with AD pathology; older age, being a man, self‐identifying as White, greater depressive symptoms, and microinfarcts were associated with infarcts; and greater loneliness (1.55 [1.16, 2.06], *p* = 0.003), infarcts, being a woman, self‐identifying as Black, and higher education was associated with microinfarcts (Table 3).

**TABLE 2:** Associations of demographic variables with loneliness in the observed and IOSW‐weighted data.

	Observed	Weighted
Intercept	2.54 [2.46, 2.63] *p* < 0.001	2.44 [2.34, 2.54] *p* < 0.001
Age	**0.02 [0.01, 0.03] *p* = 1e‐7**	**0.03 [0.03, 0.04] *p* = 5e‐18**
Sex	**−0.15 [−0.25, −0.05] *p* = 3e‐3**	5e‐3 [−0.1, 0.11] *p* = 0.93
Race	−5e‐3 [−0.2, 0.19] *p* = 0.96	−0.04 [−0.18, 0.1] *p* = 0.57
Education	**−0.02 [−0.03, −8e‐4] *p* = 0.04**	−0.01 [−0.03, 6e‐3] *p* = 0.19
Depressive symptoms	**0.13 [0.1, 0.17] *p* = 1e‐14**	**0.12 [0.08, 0.15] *p* = 6e‐11**

*Note*: Each column represents a single model. Reference group for sex was men and for race was White. Significant associations are shown in bold. IOSWs, inverse odds of selection weights.

**TABLE 3:** Associations of demographic variables and loneliness with pathology in observed and IOSW‐weighted data.

	AD pathology		Infarcts		Microinfarcts	
	Observed	Weighted	Observed	Weighted	Observed	Weighted
Intercept	1.48 [1.07, 2.04] *p* = 0.02	1.26 [0.94, 1.69] *p* = 0.12	0.45 [0.31, 0.65] *p* = 3e‐5	0.65 [0.45, 0.93] *p* = 0.02	0.25 [0.16, 0.37] *p* = 5e‐11	0.1 [0.07, 0.16] *p* = 7e‐23
Loneliness	1.19 [0.91, 1.55] *p* = 0.21	0.99 [0.72, 1.37] *p* = 0.96	1.19 [0.9, 1.56] *p* = 0.23	0.82 [0.63, 1.07] *p* = 0.14	0.86 [0.65, 1.15] *p* = 0.32	**1.55 [1.16, 2.06] *p* = 3e‐3**
AD pathology	–	–	0.76 [0.54, 1.07] *p* = 0.12	0.85 [0.59, 1.2] *p* = 0.35	1.16 [0.81, 1.65] *p* = 0.43	1.21 [0.79, 1.86] *p* = 0.39
Infarcts	0.76 [0.53, 1.07] *p* = 0.11	0.81 [0.56, 1.18] *p* = 0.27	–	–	**3.16 [2.23, 4.46] *p* = 1e‐10**	**4.2 [3.09, 5.7] *p* = 2e‐17**
Microinfarcts	1.15 [0.8, 1.64] *p* = 0.44	1.26 [0.83, 1.91] *p* = 0.29	**3.16 [2.23, 4.46] *p* = 1e‐10**	**4.28 [3.16, 5.8] *p* = 3e‐18**	–	–
Age	**1.07 [1.04, 1.1] *p* = 6e‐7**	**1.19 [1.15, 1.22] *p* = 7e‐24**	1.01 [0.98, 1.04] *p* = 0.42	**1.06 [1.03, 1.09] *p* = 2e‐5**	**1.04 [1.01, 1.07] *p* = 9e‐3**	1 [0.98, 1.03] *p* = 0.74
Sex	0.86 [0.61, 1.22] *p* = 0.4	0.83 [0.6, 1.15] *p* = 0.26	0.91 [0.63, 1.32] *p* = 0.63	**0.44 [0.31, 0.62] *p* = 8e‐6**	1.1 [0.75, 1.61] *p* = 0.62	**2.89 [2.07, 4.04] *p* = 2e‐9**
Race	1.76 [0.89, 3.51] *p* = 0.11	**2.03 [1.29, 3.21] *p* = 3e‐3**	0.72 [0.34, 1.55] *p* = 0.41	**0.58 [0.38, 0.88] *p* = 0.01**	0.93 [0.42, 2.05] *p* = 0.85	**2.23 [1.47, 3.37] *p* = 2e‐4**
Education	0.95 [0.9, 1.01] *p* = 0.11	0.97 [0.92, 1.03] *p* = 0.35	0.95 [0.9, 1.01] *p* = 0.13	0.97 [0.92, 1.02] *p* = 0.26	0.99 [0.93, 1.05] *p* = 0.63	**1.1 [1.04, 1.17] *p* = 2e‐3**
Depressive symptoms	0.89 [0.79, 1] *p* = 0.06	**1.34 [1.19, 1.51] *p* = 6e‐6**	1.01 [0.89, 1.14] *p* = 0.9	**1.15 [1.04, 1.27] *p* = 5e‐3**	1.02 [0.89, 1.15] *p* = 0.82	0.95 [0.86, 1.05] *p* = 0.31
Time between last visit and autopsy	**1.34 [1.15, 1.57] *p* = 2e‐4**	**1.27 [1.03, 1.57] *p* = 0.03**	1.04 [0.91, 1.19] *p* = 0.58	1.15 [0.92, 1.43] *p* = 0.23	1.04 [0.91, 1.19] *p* = 0.54	0.83 [0.69, 1.01] *p* = 0.07

*Note*: Each column represents a single model. Reference group for sex was men and for race was White. Significant associations are shown in bold. IOSWs, inverse odds of selection weights.

### 3.2 Loneliness, pathology, and cognition

**FIGURE 1 alz14196-fig-0001:**
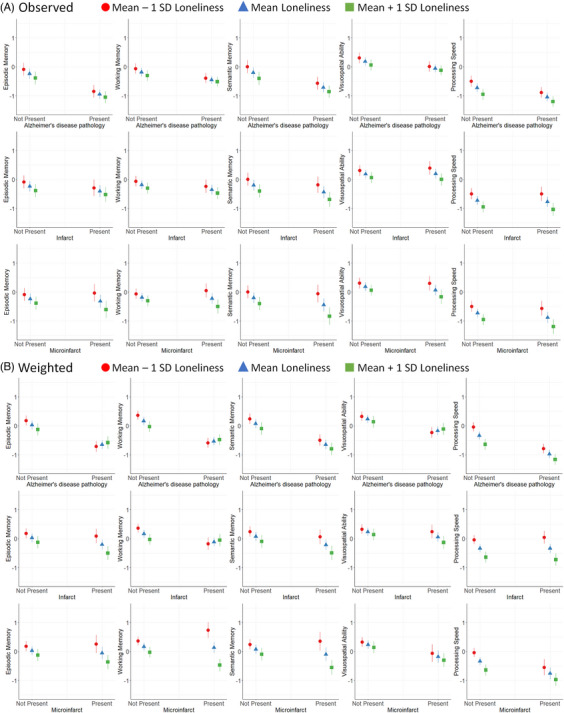
The moderating effect of loneliness with neuropathology and cognitive performance across five domains in the (A) observed and (B) IOSW‐weighted data. IOSWs, inverse odds of selection weights.

In the observed data, greater loneliness was associated with worse performance across all cognitive domains (−0.35 to −0.19, *p*’s < 0.05), adjusting for AD and cerebrovascular pathology (Table 4; Figure 1A). AD pathology was associated with worse performance across all domains; infarcts were associated with lower episodic (−0.17 [−0.34, −0.0007], *p* = 0.05), working (−0.17 [−0.31, −0.04], *p* = 0.01), and semantic memory (−0.24 [−0.41, −0.07], *p* = 0.005). Microinfarcts were associated with lower semantic memory (−0.25 [−0.42, −0.08], *p* = 0.005) and processing speed (−0.16 [−0.3, −0.01], *p* = 0.04). Loneliness did not moderate the associations between AD pathology or infarcts and any cognitive domain score. Microinfarcts had a stronger negative association with working (−0.24 [−0.47, −0.02], *p* = 0.03) and semantic memory (−0.29 [−0.57, −0.01], *p* = 0.04) at greater loneliness (Figure 1A).

**TABLE 4:** Associations of demographic variables, loneliness, pathology, and loneliness by pathology interactions on cognitive performance across five domains.

	Episodic memory		Working memory		Semantic memory		Visuospatial ability		Processing speed	
	Observed	Weighted	Observed	Weighted	Observed	Weighted	Observed	Weighted	Observed	Weighted
Intercept	−0.24 [−0.42, −0.06] *p* = 0.01	0.16 [−0.02, 0.34] *p* = 0.08	−0.18 [−0.33, −0.04] *p* = 0.02	0.29 [0.13, 0.46] *p* = 6e‐4	−0.2 [−0.38, −0.02] *p* = 0.03	0.08 [−0.08, 0.25] *p* = 0.32	0.18 [0.03, 0.33] *p* = 0.02	0.43 [0.26, 0.6] *p* = 6e‐7	−0.72 [−0.88, −0.57] *p* = 2e‐19	−0.25 [−0.42, −0.09] *p* = 3e‐3
Loneliness	−**0.23 [−0.44, −0.03] *p* = 0.03**	−**0.2 [−0.39, −8e‐3] *p* = 0.04**	−**0.19 [−0.35, −0.02] *p* = 0.03**	−**0.27 [−0.44, −0.1] *p* = 2e‐3**	−**0.31 [−0.52, −0.11] *p* = 3e‐3**	−**0.21 [−0.39, −0.02] *p* = 0.03**	−**0.19 [−0.36, −0.02] *p* = 0.03**	−0.1 [−0.28, 0.07] *p* = 0.25	−**0.35 [−0.53, −0.18] *p* = 7e‐5**	−**0.43 [−0.62, −0.25] *p* = 4e‐6**
AD pathology	−**0.71 [−0.87, −0.55] *p* = 2e‐17**	−**0.63 [−0.81, −0.46] *p* = 4e‐11**	−**0.27 [−0.39, −0.14] *p* = 6e‐5**	−**0.63 [−0.8, −0.46] *p* = 2e‐11**	−**0.52 [−0.67, −0.36] *p* = 4e‐10**	−**0.58 [−0.78, −0.37] *p* = 1e‐7**	−**0.24 [−0.37, −0.11] *p* = 4e‐4**	−**0.4 [−0.58, −0.22] *p* = 3e‐5**	−**0.32 [−0.45, −0.18] *p* = 4e‐6**	−**0.57 [−0.73, −0.4] *p* = 3e‐10**
AD pathology X Loneliness	0.07 [−0.17, 0.31] *p* = 0.56	**0.37 [0.15, 0.59] *p* = 1e‐3**	0.09 [−0.1, 0.28] *p* = 0.37	**0.4 [0.19, 0.6] *p* = 2e‐4**	0.09 [−0.15, 0.33] *p* = 0.48	0.08 [−0.13, 0.29] *p* = 0.47	0.09 [−0.11, 0.28] *p* = 0.4	0.17 [−0.04, 0.39] *p* = 0.12	0.11 [−0.1, 0.31] *p* = 0.3	0.18 [−0.03, 0.39] *p* = 0.09
Infarcts	−**0.17 [−0.34, −7e‐4] *p* = 0.05**	−**0.19 [−0.36, −0.02] *p* = 0.03**	−**0.17 [−0.31, −0.04] *p* = 0.01**	−**0.23 [−0.4, −0.05] *p* = 0.01**	−**0.24 [−0.41, −0.07] *p* = 5e‐3**	−**0.28 [−0.46, −0.1] *p* = 2e‐3**	0.01 [−0.13, 0.15] *p* = 0.87	−0.08 [−0.26, 0.1] *p* = 0.38	−0.04 [−0.19, 0.1] *p* = 0.55	0.02 [−0.17, 0.21] *p* = 0.83
Infarcts X Loneliness	0.05 [−0.21, 0.31] *p* = 0.69	−**0.33 [−0.61, −0.04] *p* = 0.03**	8e‐4 [−0.21, 0.21] *p* = 0.99	**0.32 [0.07, 0.57] *p* = 0.01**	−0.08 [−0.34, 0.18] *p* = 0.54	−**0.29 [−0.57, −0.01] *p* = 0.04**	−0.12 [−0.33, 0.1] *p* = 0.28	−0.25 [−0.52, 0.02] *p* = 0.07	−0.07 [−0.29, 0.15] *p* = 0.56	−0.26 [−0.52, 8e‐3] *p* = 0.06
Microinfarcts	−0.08 [−0.25, 0.09] *p* = 0.36	−0.14 [−0.33, 0.06] *p* = 0.17	−0.04 [−0.18, 0.1] *p* = 0.58	−0.12 [−0.31, 0.06] *p* = 0.2	−**0.25 [−0.42, −0.08] *p* = 5e‐3**	−**0.29 [−0.5, −0.07] *p* = 1e‐2**	−0.12 [−0.26, 0.02] *p* = 0.1	−**0.44 [−0.64, −0.25] *p* = 2e‐5**	−**0.16 [−0.3, −0.01] *p* = 0.04**	−**0.5 [−0.71, −0.29] *p* = 8e‐6**
Microinfarcts X Loneliness	−0.22 [−0.49, 0.06] *p* = 0.13	−0.25 [−0.51, 6e‐3] *p* = 0.06	−**0.24 [−0.47, −0.02] *p* = 0.03**	−**0.61 [−0.85, −0.37] *p* = 1e‐6**	−**0.29 [−0.57, −0.01] *p* = 0.04**	−**0.44 [−0.71, −0.18] *p* = 1e‐3**	−0.17 [−0.4, 0.06] *p* = 0.14	−0.02 [−0.28, 0.23] *p* = 0.85	−0.14 [−0.37, 0.1] *p* = 0.25	0.17 [−0.09, 0.43] *p* = 0.21
Age	−**0.02 [−0.03, −3e‐3] *p* = 0.02**	‐9e‐3 [−0.02, 3e‐3] *p* = 0.15	‐8e‐3 [−0.02, 3e‐3] *p* = 0.16	6e‐4 [−0.01, 0.01] *p* = 0.93	−**0.02 [−0.03, −5e‐3] *p* = 7e‐3**	−**0.03 [−0.04, −0.02] *p* = 3e‐6**	−**0.01 [−0.02, −3e‐3] *p* = 0.01**	‐4e‐3 [−0.02, 9e‐3] *p* = 0.57	−**0.03 [−0.04, −0.02] *p* = 8e‐7**	−**0.01 [−0.02, −9e‐4] *p* = 0.04**
Sex	**0.28 [0.11, 0.45] *p* = 1e‐3**	**0.29 [0.13, 0.44] *p* = 4e‐4**	0.06 [−0.08, 0.19] *p* = 0.4	‐1e‐3 [−0.15, 0.15] *p* = 0.99	−0.02 [−0.19, 0.15] *p* = 0.79	−0.07 [−0.23, 0.09] *p* = 0.41	−**0.29 [−0.43, −0.15] *p* = 5e‐5**	−**0.15 [−0.31, 2e‐3] *p* = 0.05**	0.1 [−0.04, 0.24] *p* = 0.17	−0.12 [−0.26, 0.02] *p* = 0.1
Race	−0.01 [−0.34, 0.32] *p* = 0.95	0.08 [−0.13, 0.29] *p* = 0.47	−0.19 [−0.46, 0.08] *p* = 0.16	−0.16 [−0.36, 0.04] *p* = 0.11	−0.06 [−0.4, 0.27] *p* = 0.7	−0.13 [−0.35, 0.09] *p* = 0.24	−**0.46 [−0.73, −0.18] *p* = 1e‐3**	−**0.44 [−0.65, −0.24] *p* = 3e‐5**	−0.22 [−0.5, 0.06] *p* = 0.13	−0.04 [−0.26, 0.17] *p* = 0.69
Education	**0.03 [6e‐3, 0.06] *p* = 0.02**	**0.08 [0.05, 0.11] *p* = 2e‐7**	**0.02 [1e‐3, 0.05] *p* = 0.04**	**0.06 [0.04, 0.09] *p* = 9e‐6**	0.03 [−2e‐3, 0.05] *p* = 0.07	**0.08 [0.05, 0.11] *p* = 2e‐8**	**0.05 [0.03, 0.07] *p* = 2e‐5**	**0.08 [0.05, 0.11] *p* = 8e‐9**	**0.04 [0.02, 0.07] *p* = 3e‐4**	**0.08 [0.05, 0.11] *p* = 8e‐9**
Depressive symptoms	0.03 [−0.02, 0.09] *p* = 0.26	−0.03 [−0.08, 0.02] *p* = 0.19	0.03 [−0.02, 0.08] *p* = 0.22	−**0.07 [−0.11, −0.02] *p* = 7e‐3**	0.04 [−0.02, 0.1] *p* = 0.16	7e‐3 [−0.05, 0.06] *p* = 0.79	‐1e‐2 [−0.06, 0.04] *p* = 0.69	−**0.12 [−0.16, −0.07] *p* = 1e‐6**	0.02 [−0.03, 0.07] *p* = 0.45	−0.03 [−0.07, 0.02] *p* = 0.28
Time between last visit and autopsy	0.04 [−0.03, 0.1] *p* = 0.25	1e‐2 [−0.11, 0.13] *p* = 0.88	0.03 [−0.02, 0.08] *p* = 0.21	0.05 [−0.04, 0.14] *p* = 0.28	**0.07 [0.01, 0.14] *p* = 0.02**	0.11 [−0.02, 0.25] *p* = 0.1	0.03 [−0.02, 0.08] *p* = 0.3	0.05 [−0.05, 0.15] *p* = 0.32	0.03 [−0.02, 0.08] *p* = 0.25	6e‐3 [−0.1, 0.11] *p* = 0.91

*Note*: Each column represents a single model. Reference group for sex was men and for race was White. Significant associations are shown in bold.

After IOSW weighting, greater loneliness was associated with worse performance in all domains (−0.43 to −0.20, *p*’s < 0.05), except visuospatial ability, adjusting for AD and cerebrovascular pathology (Table 4; Figure 1B). Microinfarcts were additionally associated with lower visuospatial ability (−0.44 [−0.64, −0.25], *p* < 0.001). In the absence of AD pathology, episodic and working memory were lower at greater loneliness, but in the presence of AD pathology, scores were similarly low regardless of loneliness (0.37 [0.15, 0.59], *p* = 0.001 and 0.40 [0.19, 0.60], *p* < 0.001, respectively; e.g., Figure 1B). Similarly, loneliness had a large negative association with working memory in the absence of infarcts, but scores were low in the presence of infarcts regardless of loneliness (0.32 [0.07, 0.57], *p* = 0.01). Loneliness strengthened the associations between infarcts and lower episodic (−0.33 [−0.61, −0.04], *p* = 0.03) and semantic memory (−0.29 [−0.57, −0.01], *p* = 0.04) as well as the associations between microinfarcts and lower working (−0.61 [−0.85, −0.37], *p* < 0.001) and semantic memory (−0.44 [−0.71, −0.18], *p* = 0.001).

## 4 DISCUSSION

This study focused on the relationship of loneliness with two of the most common pathologies underlying dementia (i.e., AD and cerebrovascular pathology), and how those pathological correlates are associated with the primary outcome for dementia risk—cognitive[Fig alz14196-fig-0001] performance. Selection bias in autopsy studies can have a substantive effect on conclusions drawn about relationships among social, biological, and cognitive constructs. Findings from models weighted to be representative of the U.S. older adult population demonstrate that loneliness was associated with the presence of microinfarcts and moderated neuropathology–cognition associations in a domain‐specific manner in older adults.

Autopsy samples are critical for understanding AD and cerebrovascular pathology, but are definitionally limited by the inclusion of only deceased individuals, which cannot capture changes over time and may only reflect end‐stage disease. Transportability methods can generalize associations between loneliness, AD and cerebrovascular pathology, and cognition from a selected sample that volunteered for brain donation. We found, as expected, that IOSW weights shifted these demographics more toward those of the U.S. population. Specifically, the weighted models added more influence to younger individuals with lower pathology, which may capture earlier disease processes.

Loneliness, pathology, and cognition were associated with different factors in the observed and IOSW weighted data. Correlates of loneliness included age, sex, education, and depression in the observed data, but were limited to age and depression once the data were weighted for representativeness in the U.S. population. This suggests that intervention strategies may need to be targeted to the needs of specific communities, but age and depressive symptoms will likely be common drivers. However, the distribution of loneliness scores did not change drastically with IOSW, suggesting that depressive symptoms, the presence of pathology, and cognitive performance are more likely to be factors related to selection into autopsy samples than loneliness.

Studies that adjust for depression and social isolation often show attenuated, but significant, effect sizes for loneliness and dementia‐related outcomes.^13^, ^46^, ^47^ Loneliness can be distressing to some (e.g., emotional loneliness), but not to others (e.g., solitude),^48^ and therefore not identical to depression or social isolation. In longitudinal HRS data, social isolation was found to be a predictor of depressive symptoms, whereas loneliness was found to be a predictor and an outcome of depressive symptoms.^49^ In longitudinal Rush data, baseline loneliness was found to be a predictor of cognitive decline, but baseline cognition was not found to be a predictor of loneliness over time.^13^ Furthermore, loneliness was found to be a predictor of incident dementia, and adjusting for the 9‐item CES‐D attenuated the effect of loneliness by 16%, whereas adjusting for loneliness attenuated the effect of depression by more than half.^13^

Although loneliness in the ROS/MAP/MARS autopsy sample was shown previously to be relatively stable over time,^13^ that may differ in the U.S. population, based on major life events (e.g., marriage, major sickness, change of residence) and differing conditions (e.g., community‐level social infrastructure). The De Jong–Gierveld scale and its modifications have shown internal consistency (Cronbach *α* = 0.78); robust associations with loss of a spouse, institutional living, and low self‐esteem; and have been used widely in older adults.^13^, ^48^ However, there may be limitations related to its validity in cognitively impaired adults.

The frequency of infarcts and microinfarcts in the observed data and the U.S. population representative data did not differ much; however, the frequency of co‐occurring AD pathology decreased from 56% to 15%. The prevalence of AD pathology after weighting (15%) may be underestimated for this age range (63–108 years of age) compared to approximately 20%–40% in other studies.^8^, ^12^, ^50^, ^51^, ^52^ Alternatively, the low frequency of AD pathology may be a result of those with a family history of AD or a greater concern for AD being more likely to volunteer for brain donation. Regardless, the change in the distribution of cerebrovascular and AD pathology may explain the additional loneliness–microinfarct associations, microinfarct–cognition associations, and AD pathology by loneliness and infarct by loneliness interactions on cognition in the IOSW‐weighted data compared with the observed data.

Greater loneliness was associated with the presence of microinfarcts, but not infarcts or AD pathology, potentially because cerebrovascular disease may be more sensitive to loneliness‐related pathways (e.g., health and health‐seeking behaviors, chronic neuroinflammation, vascular risk factors, and neuroendocrine dysfunction).^16^, ^53^ Microinfarcts and other forms of silent cerebrovascular disease may be of particular interest because they may go unnoticed clinically, compound over time, and ultimately have a larger effect on health.

There was a consistent negative association of loneliness with cognition, whereas the modifying effects of loneliness on the relationship between pathology and cognition were variable. Greater loneliness was associated with a stronger negative association between infarcts and episodic memory as well as microinfarcts and working and semantic memory, suggesting lower cognitive reserve. In contrast, AD pathology was associated with a weaker negative association between episodic memory and working memory with greater loneliness because cognitive scores were already low in the absence of AD pathology with greater loneliness. Similarly, infarcts had a weaker negative association between working memory and greater loneliness because working memory scores were already low in the absence of infarcts with greater loneliness. These results could be interpreted as loneliness being a strong driver of cognition in the context of no AD pathology or infarcts, but AD pathology and infarcts being stronger drivers of cognition when present.

Previous work found an association between greater loneliness (measured with the UCLA Loneliness Scale) and amyloid^54^, ^55^ and tau^56^ positron emission tomography (PET) burden in adults with an average age of 75 at a different period of susceptibility (e.g., preclinical AD) and/or with different measurement (e.g., continuous vs binary biomarkers, amyloid or tau vs amyloid and tau, PET vs pathology). Similar to our findings with microinfarcts, other work found that loneliness was associated with greater small vessel disease,^57^, ^58^ which may have a mechanistic link to AD pathology and dementia.^44^, ^59^, ^60^ Loneliness was associated with an increased risk for AD dementia, but not vascular dementia,^20^ despite no association with AD pathology^13^; therefore, loneliness may increase AD risk through its effect on infarcts and microinfarcts. Loneliness and social isolation were found to be associated with lower gray matter volume in regions including the hippocampus,^61^, ^62^ potentially underlying its direct effect on cognition. Social network size moderated the effect of AD pathology on semantic and working memory^47^ and the effect of neurodegeneration on language and processing speed,^15^ potentially via a cognitive reserve mechanism. In meta‐analyses, moderate heterogeneity across studies exists in study design (e.g., follow‐up length), loneliness measure, and covariates (e.g., depression, social isolation, clinical variables [cardiovascular health], behavioral variables [exercise], and genetic risk), but there are consistent associations between loneliness and dementia‐related outcomes.^29^, ^63^, ^64^, ^65^, ^66^

Limitations of the current study include cross‐sectional associations restricted to additive models of AD and cerebrovascular pathology on cognition, a singular focus on loneliness, global and binary pathological ratings, and the exploratory nature of IOSWs. It is possible that microinfarcts could increase the susceptibility to loneliness (e.g., vascular depression hypothesis^67^, ^68^) rather than loneliness leading to chronic stress and health and health‐seeking behaviors to increase susceptibility to microinfarcts. The incidence, severity, context, and trajectories of loneliness over time, as well as the reasons for loneliness, will likely be important factors to consider. This analysis focused on the two most common pathologies in AD dementia; incorporating the location, continuous measures, and potential interactions of amyloid, tau, vascular pathology, and other pathologies may provide additional insight. Within the Rush cohorts there was no requirement for brain donation in MARS and an older age of enrollment in MAP. Another potentially important difference between the Rush cohorts and HRS that may not be fully captured by the covariate balance at baseline is differential attrition (e.g., yearly follow‐ups in Rush compared to the follow‐ups every 2 years in HRS). Weighted results will require replication in measured data from representative cohorts.

These findings point to future work aimed at understanding the dependencies of loneliness on life stage and susceptibility periods for pathogenesis, and relevant community and societal factors. From a socioecological perspective, loneliness operates on the individual, interpersonal, community, and societal levels.^16^, ^69^ Social interventions directly addressing loneliness, policies to strengthen communities and their resources, and cultural changes to promote positive social engagement in real life and through technology at all ages may prevent loneliness and reduce cognitive impairment. Longitudinal data across the life course to assess the incidence, severity, and trajectories of loneliness along with the incidence, severity, and location of cerebrovascular disease, may provide insight into the additive and/or synergistic effects with AD pathology and other concurrent pathologies (e.g., other vascular pathologies beyond infarcts and microinfarcts, TAR DNA‐binding protein 43 (TDP‐43), alpha‐synuclein). Multi‐level intervention strategies should consider cerebrovascular pathology, cognitive impairment, and cognitive reserve as loneliness‐related health outcomes.

## CONFLICT OF INTEREST STATEMENT

All authors have nothing to disclose. Author disclosures are available in the supporting information.

## CONSENT STATEMENT

All participants provided informed consent and all study procedures were approved by the Rush University Institutional Review Board and performed according to the Declaration of Helsinki.

## Supporting information

Supporting Information

Supporting Information
