# Supplementary material for: Loneliness, cerebrovascular and Alzheimer's disease pathology, and cognition
Source: Alzheimers Dement. 2024 Sep 5;20(10):7113–23. doi: 10.1002/alz.14196 (PMC11485071; doi:10.1002/alz.14196)
Supplement: Supplementary file 1 — Supporting Information [file ALZ-20-7113-s001.docx]

Supplementary Materials

ROS/MAP/MARS participants were eligible for inclusion in this analysis if they were 65 years or older and agreed to autopsy, among other criteria[1, 2]. HRS participants for transportability were limited to those in the 2010 wave (when additional variables were available for harmonization) and 65 years or older (n=8469; compared to the general HRS inclusion which was 51 years and older)[3]. Harmonized variables included age, sex, race, education, married status, immigration status, work status, body mass index (BMI), diabetes status, hypertension status, alcohol intake, smoking status, depression (8-item CESD was available in HRS and the 10-item CESD in Rush was collapsed into a sum of the same 8 items (e.g., …you felt: depressed, everything you did was an effort, sleep was restless, happy, lonely, enjoyed life, sad, you could not get going), dementia rating, an orientation measure, a word list recall measure (immediate and delayed), and a verbal fluency measure. Only harmonized variables between the analytic sample and the external population can be used to calculate IOSW weights.

Separate multiple imputation models were run in each dataset, using the most available information in each study to provide the most robust imputations. We erred on the side of including as many variables as possible, as bias would be greater for models excluding important variables compared to models including extra variables. In ROS/MAP/MARS, the multiple imputation model included harmonized variables, analytic variables, and auxiliary variables to preserve associations within the sample (AD pathological rating, chronic infarcts, chronic microinfarcts, loneliness, loneliness X pathology interactions, cognitive measures, vascular pathological ratings, Lewy body pathological ratings, TDP43 pathological ratings, hippocampal sclerosis ratings; discrimination rating, social activity rating, self-reported health rating (vascular disease, stroke, medical conditions), physical activity, basic and instrumental activities of daily living, and memory complaints). In HRS, the multiple imputation model included harmonized variables and auxiliary variables to preserve associations within the sample (household income, number of health insurance plans, self-reported health rating, psychiatric problems, self-reported heart problems, self-reported memory rating, activities of daily living, total mental status summary score, telephone interview for cognitive status, immediate word recall, delayed word recall, and imputed memory score). Unless specified, missingness was below 5% in ROS/MAP/MARS (harmonized variables: 9.7% married; analytic variables: 6.2% TDP43 pathology; missing auxiliary variables: 50.1% discrimination, 46% social activity, 45.7% self-reported vascular health) and in HRS (harmonized variables: 8.8% dementia rating; auxiliary variables: 10.2% telephone interview for cognitive status, 8.5% imputed memory score). Multiple imputations were run using the ‘mice’ package in R (version 4.0.2) with 40 imputations and 10 iterations.

Covariate balance plots were used to assess the goodness of the match between the sample and external population. Covariate balance plots are shown unweighted prior to multiple imputation, unweighted after multiple imputation for 40 datasets, and weighted after multiple imputation for 40 datasets (Supplemental Figure 1). The final IOSW model included Age + Sex + Race + Education + Married Status + BMI + Diabetes + Hypertension + Smoking + Alcohol + Dementia + Memory score + Fluency Score + Depression + Age^2^ + Age^3^ + Age X Married Status + Age X Race + Age X Sex + Age X Education + Age X Alcohol + Race X Education X Alcohol. Covariate balance was assessed for all harmonized variables, regardless of whether they were included in the IOSW model to help ensure that the model does not overfit the data. The iterative process generally included (1) main effects of demographics, health variables, and cognitive variables, (2) main effects and non-linear effects of variables with large imbalances (e.g., age, married status), (3) age X demographic interactions with variables, (4) interactions with variables that differed too much from the unweighted covariate balance (e.g., education, race, alcohol), and (5) removal of non-linear and interaction terms while maintaining suitable balance. Stabilized IOSW were trimmed at the upper limit of the 99% confidence interval to avoid a single subject being weighted too strongly. The IOSW (i.e., weights from ROS/MAP/MARS to HRS) were multiplied by the HRS weights (i.e., weights from HRS to the US population) to transport results from the analytic sample to the overall US population aged 65 and older. This methodology was similarly applied in previous publications and performed as a part of the 2021 Advanced Psychometrics Methods in Cognitive Aging Research sponsored by the National Institute on Aging (R13 AG030995; PI: Dan Mungas). In Supplemental Figure 1, the IOSW model achieved suitable (<0.25 SD) covariate balance across all harmonized variables. An example interpretation for the top 3 unbalanced covariates would be that, before IOSW weighting, the analytic sample is less likely to marry, has a greater number of years of education, and older when compared to the overall US population aged 65 and older. At baseline, which was used for calculating inverse odds of selection weights, 4.3% of the Rush autopsy sample was diagnosed with dementia while 4.8% of the HRS sample had a probability of dementia greater than 0.5 (i.e., dichotomized for harmonization). At the last study visit, 25.1% of the Rush autopsy sample was diagnosed with dementia. After IOSW weighting, 14.5% of the Rush autopsy sample was diagnosed with dementia.


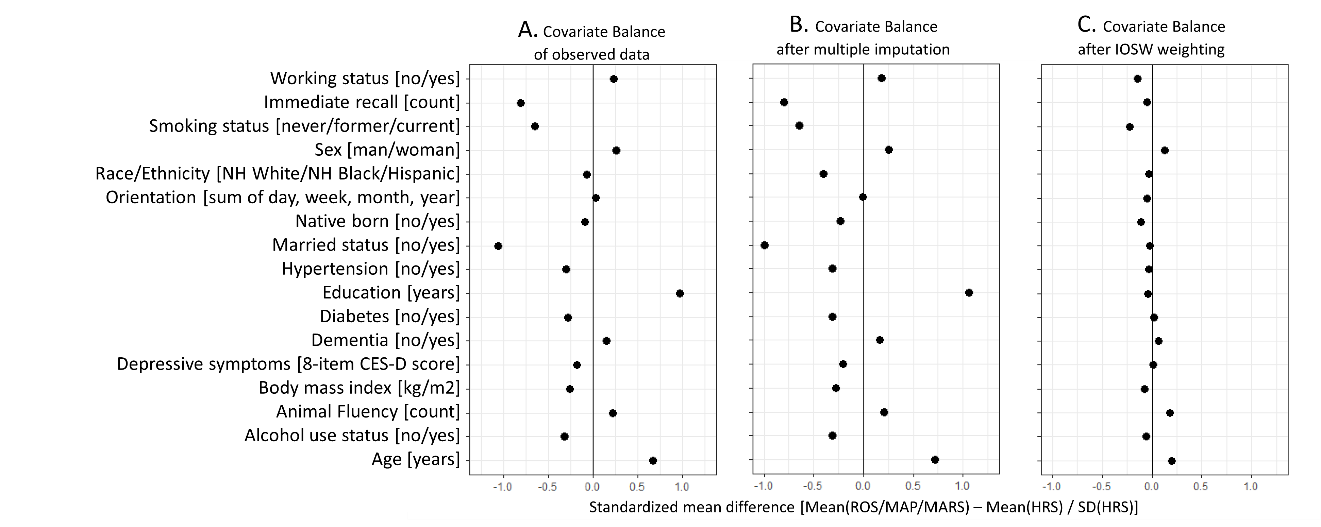


Supplemental Figure 1. Covariate balance plots for all harmonized variables (in descending alphabetical order) for the observed data (A), imputed data (B), and IOSW weighted data. Positive values indicate a greater value in the ROS/MAP/MARS sample compared to the HRS sample for continuous variables or a higher proportion of other levels compared to reference (i.e., the first level).

References

[1] Bennett DA, Buchman AS, Boyle PA, Barnes LL, Wilson RS, Schneider JAJJoAsd. Religious orders study and rush memory and aging project. 2018;64:S161-S89.

[2] L Barnes L, C Shah R, T Aggarwal N, A Bennett D, A Schneider JJCAR. The Minority Aging Research Study: ongoing efforts to obtain brain donation in African Americans without dementia. 2012;9:734-45.

[3] Sonnega A, Faul JD, Ofstedal MB, Langa KM, Phillips JW, Weir DRJIjoe. Cohort profile: the health and retirement study (HRS). 2014;43:576-85.
